# Setting Porcelain Inlays

**Published:** 1904-07

**Authors:** J. S. Bridges

**Affiliations:** Chicago.


### SETTING PORCELAIN INLAYS.

By J. S. Bridges, D. D. S., Chicago.

(Written for *The American Journal of Dental Science.*)

Setting an inlay is the simplest, but possibly the most trying of all preceding steps. Upon a trifle, perhaps, hangs the fate of a creation we have nursed through many tedious moments. Calmness, carefulness and preparation will lead to success when a lack of either might bring heart breaking disaster.

Assuming our finished inlay has previously been tried in the cavity, and the test is to our liking, we are ready to seat the restoration into the waiting cavity.

*Preparation.*—Lay out the mixing slab, chilling it if the weather is excessively warm. Place upon one corner a small heap of powder and opposte a bit of liquid, dropped from the bottle. A medium sized German silver spatula should be laid near by. Invert the inlay upon a bed of yellow beeswax, and with an orange wood stick drop the hydrofluoric acid on the upturned surface, previously observing that the margins are securely sealed to guard the glazed surface from injury. Allowing the acid to remain, possibly three minutes, wash off the water. With compressed air, alcohol and a fine pointed instrument remove every particle of powdered porcelain from the inlay.

By means of absorbent cotton rolls, linen napkins, spunk, alcohol and compressed air the cavity is isolated and thoroughly dried. The rubber dam is not advised except in rare cases; for by far too much danger is incurred from its tendency to buckle at the margins or become sticky from surplus cement, entangling the fingers or instruments; besides a few cervical cavities will permit the use of a clamp or ligatures without extreme discomfort to your patient and considerable physical exertion to yourself.

Birds-eye linen cut in squares of about six inches make convenient sized napkins. Turn down a corner about an inch then fold to the center from the right and left and a splendid and comfortable dam for the superior teeth is formed. Cotton rolls give most excellent service about the inferior teeth and for banking against the salivary ducts when the flow is over copious. Spunk is an admirable assistant for drying the cavity and may be used to advantage if left in the cavity to absorb any secretions during the mixing of the cement.

*Mixing the Cement.*—Bring the powder gradually, a small portion at a time, into the liquid, stirring one way until a thin cream is formed that just absorbs the last exudate of liquid. Take sufficient of this upon the point of the spatula to spread over the floor of the cavity and the base of the inlay. This done, continue adding powder to the mixture until the cream is so heavy you dare not add more for fear of losing the necessary adhesiveness. Place enough of this in the cavity to fill it to a generous overflow.

*Seating the Inlay.*—Quickly grasp the inlay with a pair of pliers and gently coax the filling over, but not completely, into its proper seat. Now, with another pair of pliers (the first most likely are gummed with cement) pick out a bit of spunk and press the inlay thoroughly home. The thin spread of cement by this time having nicely soaked into the tubulii of the cementum and the crevices of the porcelain, will act as a flux to the heavier cement sandwiched between and should allow the inlay to find its proper seat with but little strain. This arrangement of the cement will overcome many disasters from an insufficiently dense body the lack of which permits the fluids to soon undermine and cause an otherwise perfect restoration to fail.

A simple mix may make the seating somewhat easier but will leave little powder behind for the over abundance of liquid carries most of it away when the inlay is forced into place.

Frequently in seating interior fillings there is not enough space to readily manipulate the spunk. When such is the case a cuttle fish strip will be found of excellent service. Slip the tape between the inlay and approximal tooth and with the thumb or forefinger hold the end tight aganst some adjacent tooth upon the side the inlay has been inserted. With the other hand draw the tape taut and gently force the filling into place. To overcome the expansion of the cement during the process of the setting, the inlay should be kept under pressure for at least twenty minutes before allowing the moisture to reach it. When this is out of the question permit a large amount of cement to remain about the margins, possibly covering same with cement varnish, or melted paraffine which may be cleared off at the next appointment.

It should seldom be necessary to polish or grind any surface but the occlusal.

A smooth strip at the cervical margin is frequently beneficial, and when used should be followed by a bit of whiting placed under the strip, which will nicely bring back a safe surface to the products our best porcelain manufacturers have placed upon the market.

---

### CAVITY PREPARATION.

By Henry C. Raymond, D. D. S., Detroit, Mich.

(Written for *The American Journal of Dental Science.*)

